# Interpreting Agreement in AI-Assisted American Society of Anesthesiologists (ASA) Classification

**DOI:** 10.2196/111973

**Published:** 2026-10-21

**Authors:** Hakan Emirkadı

**Affiliations:** 1 Department of Anesthesiology and Reanimation, Sultan 2. Abdulhamid Han Training and Research Hospital, Selimiye Mh, Tıbbiye Cd, Istanbul, 34668, Türkiye, +90 532 260 9367

**Keywords:** AI, American Society of Anesthesiologists, ASA, ASA physical status, preoperative assessment, agreement, Cohen κ

We read with interest the study by Lin et al [1] evaluating a GPT-4o-powered system for preanesthetic assessment. The agreement between AI-generated and clinician-assigned American Society of Anesthesiologists (ASA) physical status was high. Two aspects of the analysis may help place this finding in clinical context.

First, ASA physical status is an ordinal classification. The report states that agreement was assessed using Cohen κ but does not specify whether weighting was applied. This distinction matters because an ASA III patient classified as ASA II and one classified as ASA I represent different degrees of disagreement. Weighted κ would allow these differences to be reflected in the agreement estimate [2]. The authors themselves note that comparisons with earlier interrater studies are affected by the use of weighted versus unweighted κ; the same distinction would be useful for their own estimate.

The ASA distribution makes this particularly relevant. Of the 600 patients, 498 (83%) were ASA II, whereas only 57 were ASA III and 6 were ASA IV. The authors provide the distribution of clinician and AI classifications, but not their patient-level cross-tabulation. We therefore cannot determine whether disagreements occurred mainly between neighboring classes or included larger underclassification of higher-risk patients. A cross-tabulation, with particular attention to ASA III and IV, together with weighted κ, would make the clinical meaning of the reported agreement clearer.

Second, the comparison with and without Taiwan National Health Insurance (NHI) data deserves further detail. The same 600 patients were evaluated under both conditions. The κ increased from 0.518 without NHI data to 0.883 with NHI data, a difference of 0.365. Our concern is not whether the two reported κ estimates differ, but how precisely the magnitude of that change was estimated. No confidence interval or other measure of uncertainty was reported for Δκ, and the authors state that this comparison was summarized descriptively. Because both estimates were obtained from the same patients, their dependence should be taken into account. Reporting uncertainty around Δκ, for example using paired resampling, would provide a more complete estimate of the incremental contribution of NHI data.

Both requests can be met with the existing dataset. Knowing where the disagreements fell, particularly among the 63 patients assigned ASA III or IV by clinicians, and how precisely the gain from NHI data was estimated, would help readers judge how far the reported agreement supports the use of this system in perioperative risk assessment.

## Abbreviations

ASA: American Society of Anesthesiologists
NHI: National Health Insurance
